# Root Endophytic Fungi Regulate Changes in Sugar and Medicinal Compositions of *Polygonum cuspidatum*

**DOI:** 10.3389/fpls.2022.818909

**Published:** 2022-03-24

**Authors:** Rui-Ting Sun, Xiang-Cao Feng, Ze-Zhi Zhang, Nong Zhou, Hai-Dong Feng, Yi-Mei Liu, Abeer Hashem, Al-Bandari Fahad Al-Arjani, Elsayed Fathi Abd_Allah, Qiang-Sheng Wu

**Affiliations:** ^1^College of Horticulture and Gardening, Yangtze University, Jingzhou, China; ^2^Shiyan Academy of Agricultural Sciences, Shiyan, China; ^3^College of Biology and Food Engineering, Chongqing Three Gorges University, Chongqing, China; ^4^Pharmacy Faculty, Hubei University of Chinese Medicine, Wuhan, China; ^5^Department of Botany and Microbiology, College of Sciences, King Saud University, Riyadh, Saudi Arabia; ^6^Department of Plant Production, College of Food and Agricultural Sciences, King Saud University, Riyadh, Saudi Arabia

**Keywords:** medicinal plants, mycorrhizal fungi, *Piriformospora indica*, polydatin, resveratrol

## Abstract

*Polygonum cuspidatum* Sieb. et Zucc is an important industrial crop because it contains a large amount of medicinal secondary metabolites (such as polydatin, resveratrol, chrysophanol, and emodin). However, it is unclear whether root endophytic fungi increase the content of secondary metabolites in the plant. This study aimed to analyze the effects of *Funneliformis mosseae* (*Fm*) and *Piriformospora indica* (*Pi*) alone or in combination on plant growth, root morphology, thirteen sugars concentrations, and six secondary metabolites (physcion, chrysophanol, emodin, aloe-emodin, polydatin, and resveratrol) concentrations of *P*. *cuspidatum*. After 11 weeks of the fungal inoculation, the roots could be colonized by *Fm* and *Pi* single or in combination, along with the higher root colonization frequency of *Fm* > *Pi* > *Fm* + *Pi* in the descending order. In addition, *Fm* and *Pi* improved plant growth performance (plant height, stem diameter, leaf number, and shoot and root biomass) and root morphology (average diameter, maximum diameter, total length, area, and volume) to varying degrees, depending on fungal inoculations, in which *Pi* displayed a relatively better effect on plant growth. Single *Fm* and *Pi* inoculation significantly increased three disaccharides (sucrose, maltose, and trehalose) accumulation, while dual inoculum (*Fm* + *Pi*) only elevated sucrose concentrations. Most monosaccharides concentrations, such as D-arabinose, D-galactose, D-sorbitol, D-fructose, glucose, and L-rhamnose were not altered or inhibited by the endophytic fungi, except the increase in L-fucose and inositol. All fungal treatments significantly increased root chrysophanol and resveratrol concentrations, while decreased aloe-emodin concentrations. In addition, single *Pi* and dual *Fm* + *Pi* increased emodin concentrations, and single *Fm* and dual *Fm* + *Pi* elevated physcion and polydatin concentrations. It was concluded that *Fm* and *Pi* promoted the growth of *P*. *cuspidatum*, and the combination of *Fm* and *Pi* was more conducive to the production of some secondary metabolites than single inoculation.

## Introduction

*Polygonum cuspidatum* Sieb. et Zucc is a perennial herb of the Polygonaceae family, and is also a commonly used medicinal plant. Its medicinal parts are the root and rhizome, and the main active medicinal components are polydatin, resveratrol, and anthraquinones (e.g., emodin methyl ether and emodin) (Leu et al., 2008). Therefore, *P*. *cuspidatum* is often used in the treatment of hepatobiliary diseases, coughs, burns, and acute bacterial dysentery. With the development of these active components, the exploitation of *P*. *cuspidatum* has been increasing, leading to a gradual increase in the resource consumption. An urgent problem is how to promote the plant growth of *P*. *cuspidatum* and to accelerate the accumulation of medicinal components (Chen et al., 2020).

Plant-associated endophytic fungi refer to the microorganisms that are present in various tissues and cells inside plants and do not cause disease symptoms in plants (Ortega et al., 2020). Endophytic fungi are distributed in the intercellular space of roots, stems, leaves, flowers, and fruits of plants, where endophytic fungi and plants can establish a mutually beneficial relationship (Gary, 2018; Wang et al., 2020). A feature of endophytic fungi is their ability to promote the growth of their host plants by facilitating the acquisition of nutrients such as nitrogen, phosphorus, iron, zinc, and potassium (Poveda et al., 2021). The secondary metabolites produced by endophytic fungi can effectively increase the resistance of the host plant against herbivores and pathogenic microbes (Ancheeva et al., 2020).

Arbuscular mycorrhizal (AM) fungi are widely present in soil of terrestrial ecosystems and have received much attention due to their positive roles in host plants (Asmelash et al., 2016). AM fungi accelerate plant growth, improve mineral nutrient absorption, and affect the accumulation of secondary products, especially in a variety of medicinal plants (Zeng et al., 2013; Mandal et al., 2015a; Welling et al., 2016; Liang et al., 2021; Sun et al., 2021). In a study by Mandal et al. (2015a), AM fungi increased leaf artemisinin accumulation in the medicinal plant *Artemisia annua*. In addition, AM fungal inoculation dramatically increased berberine, yaconine, and tetrandrine concentrations in *Phellodendron amurense* (Fan et al., 2006), monosaccharide enol and phenylpropanoid concentrations in *Ocimum basilicum* (Prasad et al., 2011), terpene concentrations in *Atractylodes macrocephala* (Lu et al., 2011), flavonoid concentrations in *Astragalus propinquus* (He et al., 2009) and *Poncirus trifoliata* (Chen et al., 2017), polyphyllin I, II, and VII concentrations in *Paris polyphylla* var. *yunnanensis* (Li et al., 2021), artemisinin (sesquiterpene) concentrations in *Artemisia annua* (Mandal et al., 2015b), and stevioside (diterpene) concentrations in *Stevia rebaudiana* (Mandal et al., 2015a). These results indicated that AM fungi accelerate the synthesis of bioactive secondary metabolites of industrial plants.

*Piriformospora indica* (*Pi*) is an endophytic fungus, which can be cultured *in vitro* on an artificial medium (Varma et al., 1999), along with positive effects on promoting plant growth, inducing systemic resistance, and improving nutrient uptake in plants (Oelmüller et al., 2009; Yang et al., 2020). In addition, the colonization by *Pi* distinctly stimulated the accumulation of gel and total phenol in *Aloe vera* (Sharma et al., 2014), polysaccharides in *Dendrobium officinale* (Xu et al., 2021), and asiaticoside in *Centella asiatica* (Satheesan et al., 2012), suggesting that the endophytic fungus is also capable of accelerating the production of medicinal components. Sabra et al. (2018) reported that *Pi* and *Rhizophagus irregularis* (an AM fungus), single or in combination, accelerated biomass production of *Ocimum basilicum* plants grown in contaminated soils. Single AM fungus or *Pi* showed positive benefits, but it is unclear whether the combination of both AM fungus and *Pi* further promotes the content of medicinal components in host plants, although the combination of *Pi* and AM fungus did not change the concentration of essential oils in *O*. *basilicum* after 5 weeks of inoculation (Sabra et al., 2018).

In addition, some of sugars, such as trehalose, are used in medicines (Ohtake and Wang, 2011). It is well known that AM fungi primarily utilize glucose, although small amounts of sucrose and fructose are also absorbed by intraradical hyphae of AM fungi (Roth and Paszkowski, 2017). Hexose absorbed by AM fungi is transformed into trehalose and glycogen, which are translocated for fungal metabolism or storage (Bago et al., 2000). Similarly, *Pi* also takes up carbohydrates for its growth and survival, and a high-affinity hexose transporter, *PiHXT5*, with broad substrate specificity was identified from *Pi* (Rani et al., 2016; Raj et al., 2021). Therefore, changes of root sugar components can reveal potential mechanisms of symbiosis between endophytic fungi and plants.

Since AM fungi and *Pi* collectively improve the growth of host plants and the content of medicinal components, the aim of this study was to investigate the effects of AM fungi and *Pi* singly or in combination on plant growth, sugar accumulation, and contents of major medicinal components of *P*. *cuspidatum*.

## Materials and Methods

### Fungal Inocula

An AM fungus, *Funneliformis mosseae* (*Fm*) (Nicol. & Gerd.) Gerdemann & Trappe (BGC XZ02A), was supplied by the Bank of Glomeromycota in China (BGC) (Beijing, China). The fungal strain was isolated from the rhizosphere of *Incarvillea younghusbandii* Sprague in Dangxiong, Tibet, China, and proliferated by *Trifolium repens* as the host plant under potted conditions for 3 months under the condition of 900 μmol/m^2^/s photon flux density, 28°C/22°C day/night temperature, and 68% relative air humidity. The above-ground part was removed, and the roots and growth substrates were collected and air-dried for 5 days. Then the roots were cut and mixed thoroughly with the growth substrate as the fungal inoculum, in which 23 spores/g inoculum were found after wet sieving-sucrose centrifugation method (Daniels and Skipper, 1982).

*Piriformospora indica* was kindly supplied by Professor Zhi-Hong Tian (School of Life Sciences, Yangtze University) and propagated as per the protocol described by Yang et al. (2021). After 7 days of dark culture at 30°C in solid potato dextrose agar medium, a 5 mm × 5 mm mycelium block was cut and inoculated into potato dextrose broth medium for proliferation under the condition of 150 r/min, 30°C, and dark culture for 7 days. Spore suspensions were collected, mixed with distilled water in a ratio of 1:20, and determined colorimetrically at 600 nm, reaching the concentration of 2.85 × 10^8^ CFU/ml.

### Plant Culture

Seeds of *P*. *cuspidatum* were surface sterilized with 75% ethanol solution for 10 min, washed three times with distilled water, and sown in the autoclaved mixture of soil and sand (3:1, v/v). A month later, four-leaf-old seedlings were transplanted into a plastic pot (12 cm × 18 cm), supplied with 1.5 kg autoclaved mixture of soil and sand (5:3, v/v). The physicochemical properties of the mixed growth substrate were pH 7.2, Bray-P 10.05 mg/kg, ammonium nitrogen 108.45 mg/kg, nitrate nitrogen 112.46 mg/kg, and available potassium 59.21 mg/kg. At the time of transplanting, inoculation treatments of endophytic fungi were performed. In this case, the single *Fm* treatment was inoculated with 100 g of mycorrhizal fungal inoculum, the single *Pi* was applied with 30 ml of spore suspension, and the double inoculation of *Fm* + *Pi* was carried out with the application of both 100 g of *Fm* inoculum and 30 ml of spore suspension of *Pi*. The control group received equal volume of autoclaved spore suspension of *Pi* and 100 g of autoclaved inoculum of *Fm*, plus 2 ml filtrates (30 μm) of *Fm* inoculum. After endophytic fungal inoculation, these treated plants were grown in a climatic chamber with the same environmental condition as described in above sub-section. Fungal inocula for AM fungal propagation from July 18 to October 3, 2020.

### Experimental Design

The experiment consisted of two factors: inoculation with and without *Fm* and inoculation with and without *Pi*. A total of four treatments were arranged: (i) the control without any inoculation of endophytic fungi (control); (ii) single inoculation with *Fm* (*Fm*); (iii) single inoculation with *Pi* (*Pi*); and (iv) double inoculation with both *Fm* and *Pi* (*Fm* + *Pi*). Each treatment was replicated five times, with two seedlings planted in each pot, and each replication consisted of two pots, for a total of 40 pots.

### Measurements of Plant Growth and Root Fungal Colonization Frequency

After 11 weeks of endophytic fungal inoculations, the experiment was ended. Plant height, stem diameter, and leaf number per plant were determined before harvested. Then, shoots and roots were separated, and their fresh weight was measured by an electronic balance. Roots were scanned using an Epson Scanner (J221A, Jakarta Selatan, Indonesia), followed by a WinRHIZO professional software (2007v; Regent Instruments Inc., Quebec, QC, Canada) for the analysis of root morphological parameters (e.g., total length, surface area, projected area, and volume).

Root fungal structure was stained according to the protocol described by Yang et al. (2021). The 1-cm long root segments were cleared with 10% KOH solution for 100 min at 95°C, bleached with 10% hydrogen peroxide for 15 min, acidified with 0.2 mol/L hydrochloric acid for 1 h, and finally stained with 0.05% (w/v) trypan blue in lactophenol for 3 min. After microscopic observation, the root fungal colonization frequency was expressed as the percentage of the number of fungal colonized root segments over the total number of observed root segments (Yang et al., 2021).

### Measurements of Root Sugar Concentrations

The concentration of root sugars was determined by Gas Chromatography–Mass Spectrometry (GC–MS). The 1 g of root samples was freeze-dried and ground to the powder form using a grinder (MM 400, Retsch, Haan, Germany). The 20 mg powder was incubated with 500 μl of the extraction solution (methanol:isopropanol:distilled water, 3:3:2, v/v/v), vortexed for 3 min, sonicated for 30 min in an ice bath, and centrifuged for 3 min at 14,000 *g* at 4°C. A 50 μl of the supernatant was mixed well with 20 μl of internal standard, which was blow-dried by nitrogen and lyophilized. The derivative solution was obtained by adding 100 μl pyridinium methoxide (15 mg/ml) and incubating at 37°C for 2 h, followed by adding 100 μl bstfa and incubating at 37°C for 30 min. The solution was diluted with hexane and stored in a brown injection vial for GC–MS (7890B-7000D, Agilent Technologies, Inc., Santa Clara, CA, United States). Helium was used as the carrier gas, at a flow rate of 1 ml/min. The injection volume was 3 μl. The temperature was maintained at 170°C for 2 min, then increased to 240°C at 10°C/min, to 280°C at 5°C/min, and to 310°C at 25°C/min, and finally held at 310°C for 4 min. All samples were analyzed as per selective ion monitoring mode. The injector inlet and transfer line temperature was 250 and 240°C, respectively.

### Measurements of Six Medicinal Components in Roots

The main medicinal components of *P*. *cuspidatum* roots such as polydatin, resveratrol, aloe-emodin, emodin, chrysophanol, and physcion were assayed by high-performance liquid chromatography (HPLC). The 0.1 g of dried root samples was mixed with 10 ml of 80% methanol, sonicated for 30 min, and then centrifuged at 4,000 *g* for 10 min. The supernatant was further filtered through a 0.22 μm filter membrane to obtain the test solution for analysis by HPLC (LC-20AT, Shimadzu, Tokyo, Japan). The determination was performed on an Agela Venusil XBP C18(L) column (4.6 mm × 250 mm, 5 μm) with the mobile phases of acetonitrile (A phase) and 0.1% formic acid (B phase) at a flow rate of 1.0 ml/min. The injection volume was 10 μl, the detection wavelength was 290 nm, and the column temperature was 40°C. The linear gradient elution program was as follows: 0-10 min, 15–20% A phase; 10–23 min, 20–25% A phase; 23–27 min, 25–43% A phase; 27–36 min, 43–50% A phase; 36–47 min, 50–68% A phase; and 47–65 min, 68–78% A phase.

### Statistical Analysis

The experimental data were analyzed by two-way analysis of variance with SAS Software 8.1v (SAS Institute Inc., Cary, NC, United States). Duncan’s multiple range test at the 0.05% level was utilized to compare the significant differences between the treatments. The significance of the interaction between *Fm* and *Pi* inoculation was carried out on SAS Software 8.1v. The graphing was done using the SigmaPlot 10.0v (Systat Software, Inc., San Jose, CA, United States).

## Results

### Root Fungal Colonization Frequency

**FIGURE 1:**
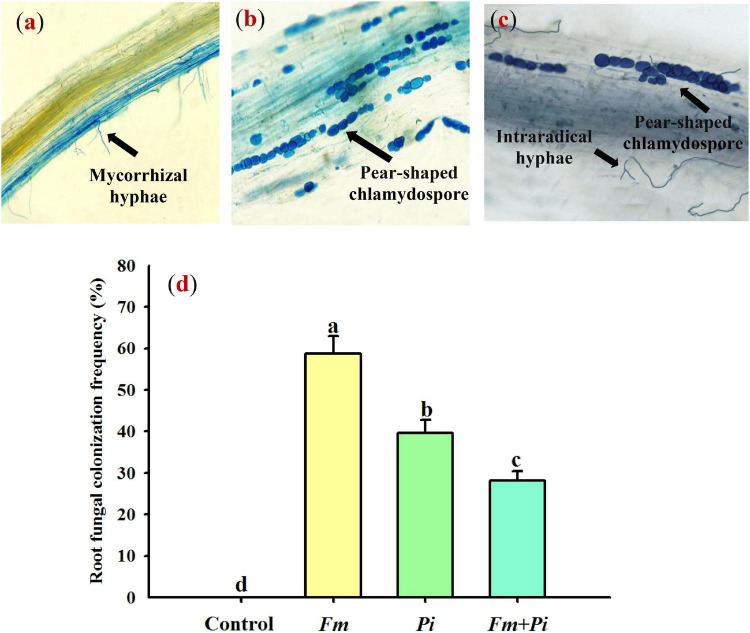
Root fungal colonization of *Polygonum cuspidatum* Sieb. et Zucc by *Funneliformis mosseae* (*Fm*) and *Piriformospora indica* (*Pi*) single or in combination. **(a)** *Fm*-inoculated roots; **(b)** *Pi*-inoculated roots; **(c)** roots inoculated with *Fm* and *Pi* in combination (*Fm* + *Pi*); **(d)** changes in root fungal colonization frequency.

In this study, *Fm* and *Pi* single or in combination infected roots of *P*. *cuspidatum*, varied from 28 to 59% of root fungal colonization frequency (Figures 1a–d). A large number of transparent chlamydospores were observed in the root of *Pi*-inoculated plants (Figure 1b); intraradical mycelia were abundant in the root of *Fm*-inoculated plants (Figure 1a); intraradical mycelia and transparent pear-shaped chlamydospores were collectively shown in the root of *Fm* + *Pi*-inoculated plants (Figure 1c). Significantly (*p* < 0.05) higher root fungal colonization frequency was listed as the trend of *Fm* > *Pi* > *Fm* + *Pi* in the decreasing order (Figure 1d). A significant (*p* < 0.05) interaction between *Fm* and *Pi* inoculation appeared in root fungal colonization frequency (Table 1).

**TABLE 1:** Significance of selective variables between *Fm* inoculation and *Pi* inoculation.

Variables	*Fm*	*Pi*	*Fm* × *Pi*
Root fungal colonization frequence	0.0269	<0.0001	<0.0001
Aloe-emodin	0.0004	<0.0001	<0.0001
Chrysophanol	<0.0001	<0.0001	<0.0001
Emodin	<0.0001	<0.0001	<0.0001
Physcion	0.6035	<0.0001	<0.0001
Polydatin	<0.0001	<0.0001	<0.0001
Resveratrol	<0.0001	<0.0001	<0.0001

### Changes in Plant Growth Performance

**FIGURE 2:**
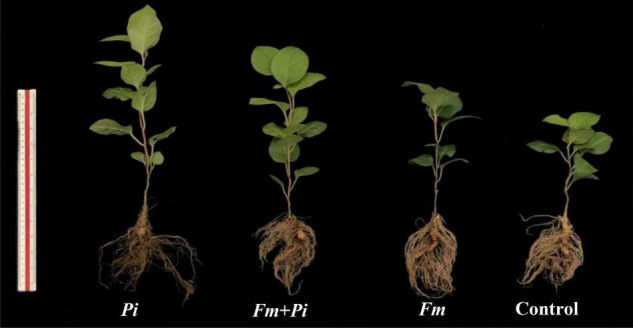
Plant growth responses of *Polygonum cuspidatum* Sieb. et Zucc to *Funneliformis mosseae* (*Fm*) and *Piriformospora indica* (*Pi*) single or in combination.

Endophytic fungi improved plant growth performance of *P*. *cuspidatum*, dependent on the species of endophytic fungi (Table 2 and Figure 2). Compared with the control, *Pi* inoculation significantly (*p* < 0.05) increased plant height, stem diameter, leaf number, shoot and root biomass by 90, 30, 11, 47, and 14%, respectively, whereas *Fm* inoculation significantly increased plant height and shoot biomass by 31 and 24%, respectively, and the double inoculation of *Fm* and *Pi* significantly increased plant height, stem diameter, and shoot biomass by 61, 30, and 47%, respectively. A significant (*p* < 0.05) interaction between *Fm* and *Pi* inoculation appeared in plant height, leaf number, and shoot biomass (Table 2).

**TABLE 2:** Effects of *Funneliformis mosseae* (*Fm*) and *Piriformospora indica* (*Pi*) single or in combination on plant growth performance of *Polygonum cuspidatum* Sieb. et Zucc.

Treatments	Plant height (cm)	Stem diameter (mm)	Leaf number (num./plant)	Shoot biomass (g FW/plant)	Root biomass (g FW/plant)
Control	14.00 ± 1.00c	2.29 ± 0.26c	11.0 ± 1.0b	3.19 ± 0.36d	5.41 ± 0.43b
*Fm*	18.40 ± 1.34b	2.45 ± 0.19bc	11.6 ± 1.1b	3.96 ± 0.63c	5.61 ± 0.46b
*Pi*	25.60 ± 2.53a	2.81 ± 0.34ab	15.0 ± 1.6a	5.77 ± 0.56a	6.54 ± 0.68a
*Fm* + *Pi*	22.50 ± 3.79a	2.98 ± 0.30a	12.2 ± 0.8b	4.70 ± 0.38b	6.18 ± 0.63ab
**Significance**					
*Fm*	<0.0001	0.0006	0.0005	0.0005	0.0036
*Pi*	0.5580	0.2201	0.0522	0.8828	0.7653
*Fm* × *Pi*	0.0033	1.0000	0.0051	0.0252	0.2732

*Data (means ± SD, n = 5) followed by different letters in the column indicate significant differences (p < 0.05).*

### Changes in Root Morphology

Inoculation with endophytic fungi improved root morphology of *P*. *cuspidatum* to some extent (Table 3). Compared with the non-inoculated control, *Fm* inoculation significantly increased root average diameter, maximum diameter, total length, projected area, and volume by 97, 72, 23, 21, and 141%, respectively; *Pi* inoculation significantly increased root maximum diameter, total length, projected area, surface area, and volume by 45, 62, 22, 45, and 98%, respectively; double *Fm* + *Pi* inoculation also significantly increased root average diameter, maximum diameter, projected area, and volume by 49, 40, 17, and 34%, respectively. There was a significant (*p* < 0.05) interaction between *Fm* and *Pi* inoculation for root average diameter, maximum diameter, total length, projected area, surface area, and volume (Table 3).

**TABLE 3:** Effects of *Funneliformis mosseae* (*Fm*) and *Piriformospora indica* (*Pi*) single or in combination on root morphology of *Polygonum cuspidatum* Sieb. et Zucc.

Treatments	Average diameter (mm)	Maximum diameter (mm)	Total length (cm)	Projected area (cm^2^)	Surface area (cm^2^)	Volume (cm^3^)
Control	2.30 ± 0.16c	8.42 ± 0.66c	142.04 ± 10.46c	10.49 ± 0.68b	12.42 ± 0.85b	0.92 ± 0.19d
*Fm*	4.52 ± 0.36a	14.48 ± 1.54a	174.31 ± 15.13b	12.71 ± 1.02a	13.43 ± 0.97b	2.22 ± 0.22a
*Pi*	2.30 ± 0.21c	12.22 ± 2.15ab	230.14 ± 22.57a	12.77 ± 1.20a	17.99 ± 1.57a	1.82 ± 0.15b
*Fm* + *Pi*	3.42 ± 0.31b	11.80 ± 1.42b	153.14 ± 15.81bc	12.32 ± 0.98a	13.69 ± 1.14b	1.23 ± 0.17c
**Significance**						
*Fm*	0.0018	0.4807	0.0016	0.0797	0.0003	0.5921
*Pi*	<0.0001	0.0032	0.0193	0.0982	0.0152	0.0022
*Fm* × *Pi*	0.0017	0.0012	<0.0001	0.0189	0.0007	<0.0001

*Data (means ± SD, n = 5) followed by different letters in the column indicate significant differences (p < 0.05).*

### Changes in Concentrations of Root Sugar Components

Endophytic fungi inoculation showed different response patterns to root sugar components (Table 4). Compared with the control, *Fm* inoculation significantly increased root inositol, L-rhamnose, L-fucose, maltose, sucrose, trehalose, and xylitol concentrations by 34, 20, 85, 98, 20, 426, and 99%, respectively, while significantly decreased D-sorbitol, D-fructose, glucose, and lactose concentrations by 19, 27, 37, and 43%. *Pi* inoculation significantly elevated root inositol, L-fucose, maltose, sucrose, and trehalose concentrations by 29, 75, 61, 46, and 145%, while reduced D-arabinose, D-galactose, D-sorbitol, D-fructose, and glucose concentrations by 10, 9, 25, 28, and 34%, respectively. Dual *Fm* + *Pi* inoculation also significantly increased inositol, L-fucose, maltose, sucrose, trehalose, and xylitol concentrations by 47, 50, 15, 25, 39, and 20%, whereas reduced D-arabinose, D-galactose, D-sorbitol, D-fructose, and glucose concentrations by 32, 40, 24, 64, and 53%, respectively. Root lactose was observed only in the plants treated by the control and single *Fm*, displaying 43% significantly lower in the *Fm*-inoculated plants than in the control plants. *Fm* inoculation and *Pi* inoculation significantly (*p* < 0.05) interacted with each other on D-galactose, glucose, L-fucose, xylitol, lactose, maltose, sucrose, and trehalose.

**TABLE 4:** Effects of *Funneliformis mosseae* (*Fm*) and *Piriformospora indica* (*Pi*) single or in combination on contents of root sugar components of *Polygonum cuspidatum* Sieb. et Zucc.

Treatments	Monosaccharides									Disaccharides			
	D-Arabinose (μg/g)	D-Galactose (μg/g)	D-Sorbitol (μg/g)	D-Fructose (mg/g)	Glucose (mg/g)	Inositol (mg/g)	L-Rhamnose (μg/g)	L-Fucose (μg/g)	Xylitol (μg/g)	Lactose (μg/g)	Maltose (μg/g)	Sucrose (mg/g)	Trehalose (μg/g)
Control	6.33 ± 0.58a	164.33 ± 9.29a	5.33 ± 0.57a	2.94 ± 0.18a	5.10 ± 0.46a	0.86 ± 0.05c	5.67 ± 0.58ab	6.67 ± 0.71c	1.67 ± 0.58b	2.33 ± 0.80a	27.33 ± 2.52c	26.97 ± 1.37c	62.00 ± 6.08c
*Fm*	6.00 ± 0.50a	162.67 ± 11.68ab	4.33 ± 0.58ab	2.14 ± 0.13b	3.22 ± 0.23b	1.15 ± 0.09b	6.44 ± 0.77a	12.33 ± 1.53a	3.33 ± 0.53a	1.33 ± 0.33b	54.00 ± 5.29a	32.23 ± 3.14b	326.00 ± 31.61a
*Pi*	5.67 ± 0.52b	149.00 ± 13.11b	4.00 ± 0.75b	2.11 ± 0.17b	3.36 ± 0.33b	1.11 ± 0.06b	5.33 ± 0.58ab	11.67 ± 1.01ab	1.67 ± 0.29b	N/A	44.00 ± 4.58b	39.27 ± 2.53a	151.67 ± 15.50b
*Fm* + *Pi*	4.33 ± 0.29c	98.00 ± 7.26c	4.05 ± 0.26b	1.05 ± 0.10c	2.41 ± 0.15c	1.26 ± 0.07a	4.50 ± 0.66b	10.00 ± 1.09b	2.00 ± 0.55b	N/A	31.33 ± 3.06c	33.80 ± 1.39b	86.33 ± 8.08c
**Significance**													
*Fm*	0.0031	0.0002	0.0395	<0.0001	0.0001	0.325	0.0165	0.0738	0.0495	<0.0001	0.2323	0.0007	0.0001
*Pi*	0.0176	0.0026	0.1868	<0.0001	<0.0001	0.2447	0.9383	0.015	0.0085	0.0312	0.0167	0.9402	<0.0001
*Fm* × *Pi*	0.1114	0.0037	0.1492	0.1531	0.033	0.711	0.0651	0.0005	0.0495	0.0312	<0.0001	0.0032	<0.0001

*Data (means ± SD, n = 3) followed by different letters in the column indicate significant differences (p < 0.05). N/A, no detected corresponding sugar.*

### Changes in Contents of Root Medicinal Components

**FIGURE 3:**
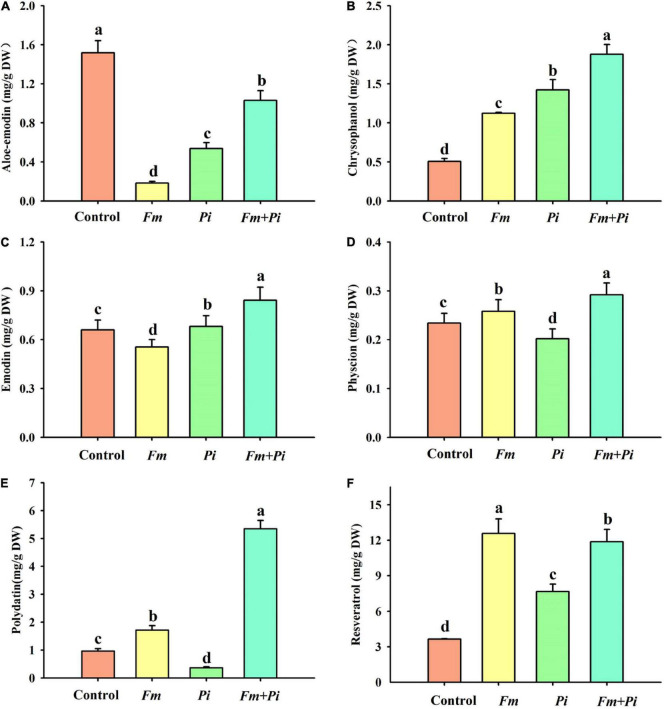
Effects of *Funneliformis mosseae* (*Fm*) and *Piriformospora indica* (*Pi*) single or in combination on root aloe-emodin **(A)**, chrysophanol **(B)**, emodin **(C)**, physcion **(D)**, polydatin **(E)**, and resveratrol **(F)** contents of *Polygonum cuspidatum* Sieb. et Zucc. Data (means ± *SD*, *n* = 3) followed by different letters above the bars indicate significant (*p* < 0.05) differences between treatments.

Six medicinal components such as aloe emodin, chrysophanol, emodin, physcion, polydatin, and resveratrol were successfully detected in the root (Figures 3A–F). Endophytic fungi significantly increased root chrysophanol and resveratrol concentrations: 122, 181, and 271% higher by *Fm*, *Pi*, and *Fm* + *Pi* in the chrysophanol (Figure 3B) and 245, 110, and 226% higher by *Fm*, *Pi*, and *Fm* + *Pi* in the resveratrol (Figure 3F). *Fm*, *Pi*, and *Fm* + *Pi* significantly decreased root aloe-emodin concentrations by 88, 65, and 32%, respectively, compared with the control (Figure 3A). However, endophytic fungi displayed diverse changes in physcion, emodin, and polydatin concentrations. Compared with the control, *Fm* and *Fm* + *Pi* significantly increased root physcion by 10 and 25% and polydatin concentrations by 78 and 453%, respectively (Figures 3D,E); *Pi* significantly reduced root physcion and polydatin concentrations by 14 and 62%, respectively. *Pi* and *Fm* + *Pi* significantly increased root emodin concentrations by 3.3 and 28%, respectively, while *Fm* significantly decreased root emodin concentrations by 16%, as compared with the control treatment (Figure 3C). The interaction study showed that *Fm* and *Pi* inoculation significantly (*p* < 0.05) interacted with each other on all the six medicinal components (Table 1).

## Discussion

In this experiment, we observed that *Fm* and *Pi* single or in combination could infect the root of *P*. *cuspidatum* Sieb. et Zucc, along with the decreasing order of *Fm* > *Pi* > *Fm* + *Pi* in the root fungal colonization frequency. Similar result was observed in trifoliate orange (*Poncirus trifoliata*) seedlings colonized by *Fm* and *Pi* single or in combination (Yang et al., 2021). Therefore, it was suggested that the roots of *P*. *cuspidatum* were preferentially colonized by *Fm*, but the *Fm* + *Pi* inoculation inhibited each other. The decrease in root fungal colonization frequency is explained by the fact that *Fm* and *Pi* compete jointly for the same colonization sites. On the other hand, *Pi* mainly colonized root cortical cells (Deshmukh et al., 2007), accelerating the programmed cell death and thus reducing the colonization sites for *Fm*.

Arbuscular mycorrhizal fungi are able to form a symbiotic relationship with most terrestrial plants and help host plants to absorb water and nutrients, which in turn improves the growth of the host plant (Smith and Read, 2008). In the present study, *Fm* significantly improved plant height and shoot biomass, suggesting a positive benefit on *P*. *cuspidatum*. On the other hand, *Pi* also significantly improved plant height, stem diameter, leaf number, and shoot and root biomass, which is consistent with the findings of Bagde et al. (2014) in the medicinal plant *Aristolochia debilis*. Moreover, the promoted effect of *Pi* was significantly higher on plant growth than that of *Fm*, suggesting a higher potential of *Pi* on *P*. *cuspidatum* than *Fm*. This may be due to the fact that *Pi* promoted the initial growth of plants mainly by accelerating the growth and development of roots (Waller et al., 2005) and producing indole-3-acetic acid and indole-3-lactate (Hilbert et al., 2012). In contrast, the growth-promoting effect of *Fm* was significantly higher than that of *Pi* on *Poncirus trifoliata* (Yang et al., 2021), suggesting a host-dependent feature. Dual *Fm* + *Pi* inoculation also significantly increased stem diameter, plant height, and shoot biomass of *P*. *cuspidatum*, and the increase amplitude was between single *Fm* and single *Pi* treatment, which may be related to the change in root fungal colonization frequency.

In our study, root morphology was partly improved by the root endophytic fungi, showing a significant increase in maximum diameter, projected area, and volume. Among the three fungal inoculations, *Fm* presented the greatest positive effect on increasing maximum diameter, volume, and average diameter, and *Pi* presented the greatest positive benefit in total length, surface area, and projected area. This implies that the improvement of root morphology in *P*. *cuspidatum* is dependent on the fungal species used. The result is in agreement with the findings of Wu et al. (2012) inoculating *Fm* in *Citrus tangerina*, Huang et al. (2020) inoculating *Fm* in *Juglans regia*, and Sharma and Agrawal (2013) inoculating *Pi* in the medicinal plant *Aloe vera*. Thus, endophyte-regulated improvement of root morphology was observed in cutting and seedlings (Verma et al., 2021). Better root morphology in inoculated plants versus non-inoculated plants enables to obtain more soil nutrients (Verma et al., 2021), which is one reason for the improved growth of inoculated plants. *Pi* is known to synthesize hormones (e.g., auxins) and release it into plant tissues (Hilbert et al., 2012; Bhatnagar et al., 2019), thus, stimulating plant growth and root morphogenesis. *Fm* also increased auxins and polyamine levels in host plants (Wu et al., 2012; Zhang F. et al., 2019). In addition, endophytic fungi also trigger the interaction of nutrients (such as P, N, and K) and reactive oxygen species/antioxidants, which transmit signals to induce the expression of root developmental genes for the improvement of root development (Verma et al., 2021). Therefore, the improvement of root morphology under endophytic fungi inoculation conditions is closely related to the regulation of root hormone levels, especially auxins.

In this study, 13 sugar components were measured in the root of *P*. *cuspidatum*, such as 9 monosaccharides (D-arabinose, xylitol, L-rhamnose, L-fucose, D-fructose, D-galactose, glucose, D-sorbitol, and inositol) and 4 disaccharides (sucrose, lactose, maltose, and trehalose). Our results revealed that *Fm* and *Pi* single and in combination significantly increased disaccharides contents, except lactose, compared with the control treatment. AM fungi mainly use glucose from sucrose cleavage as a substrate for their respiration (Zakaria Solamanand and Saito, 1997; Pfeffer et al., 1999; Wu et al., 2016). We also found that inoculation with *Fm* and *Pi*, single and in combination, significantly increased root sucrose concentration, while dramatically decreased root glucose and D-fructose, indicating that endophytic fungus-inoculated roots form a stronger sucrose pool than the non-inoculated ones. Meng et al. (2021) also reported the increase in root sucrose concentrations of trifoliate orange seedlings by *Fm* and *Pi* single and in combination. Therefore, the endophytic fungus-colonized plants accumulated more sucrose to provide adequate substrates for the fungus’ respiration in roots. AM fungi are known to use glucose primarily, and Pi also uses carbohydrates (Bago et al., 2000; Raj et al., 2021). The present study also observed that endophytic fungal colonization significantly reduced root glucose and D-fructose concentrations, indicating that both fungi utilized hexose. Moreover, the reduced effect of dual *Fm* + *Pi* inoculation on glucose and D-fructose was more prominent than that of single *Fm* and *Pi* inoculation, indicating that the loss of hexose in roots was exacerbated by dual *Fm* + *Pi* inoculation.

Trehalose is a disaccharide of glucose, which has been reported in various organisms and applied in medicines, foods, and cosmetics (Ohtake and Wang, 2011). In the life of AM fungi, glycogen and trehalose are the main carbohydrate sources, derived from hexose conversion (Sharma et al., 2020). Spores of AM fungi (e.g., *Glomus intraradix*) contain a certain amount of trehalose, accounting for 0.06–1.6% of spore weight (Becard et al., 1991). Our study showed the significant increase in root trehalose by single *Fm* and *Pi* inoculation, and the increased effect of single *Fm* was greater than that of single *Pi*. Similar result was reported in *Tagetes tenuifolia* and *Glycine max* colonized by *Fm* (Schubert et al., 1992). This showed that root-associated endophytic fungi strongly stimulate the production of trehalose in roots, and *Fm* has a more prominent advantage than *Pi*. Accumulation of trehalose in plants infected by endophytic fungi is associated with the stimulation of terpene synthase activity and the inhibition of trehalose activity by endophytic fungi (Garg and Pandey, 2016). Dual inoculation of *Fm* and *Pi* did not change the trehalose content of *P*. *cuspidatum* roots, which may be caused by the simultaneous competition of two fungi for trehalose. This suggests that a single endophytic fungus has a strong promoting effect on trehalose production in *P*. *cuspidatum* roots. In addition, D-arabinose, D-galactose, D-sorbitol, D-fructose, glucose, and L-rhamnose concentrations were suppressed or not altered after inoculation with the endophytic fungi single or in combination, except the increase in L-fucose and inositol by *Fm*, *Pi*, and *Fm* + *P* and xylitol by only *Fm*, indicating that these endophytic fungi did not affect or even inhibited the concentration of most monosaccharides in *P*. *cuspidatum*. More work needs to be carried out around the changes of endophytic fungi on monosaccharides and disaccharides of *P*. *cuspidatum*.

*Pediomelum cuspidatum* mainly contains stilbenes (e.g., resveratrol, polydatin, and their derivatives), anthraquinones (e.g., emodin, polydatin, rhein acid, and chrysophanol), flavonoids (polydatin, quercetin, canthol, and apigenin), tannins, and polysaccharides (Chen et al., 2020; Zhang et al., 2020). In this study, polydatin, resveratrol, aloe-emodin, emodin, chrysophanol, and physcion were identified in roots of *P*. *cuspidatum* and also affected by root endophytic fungi. Among them, resveratrol concentrations were significantly increased by *Fm* and *Pi* single or in combination, with the highest effect after *Pi* inoculation. Resveratrol is one of the constituents of *P*. *cuspidatum* with anti-inflammatory, anti-cancer, anti-HIV, anti-fungal, and anti-oxidant activities and has become an important drug to be exploited (Lee et al., 2015). Wang D. (2008) also observed significantly higher resveratrol concentrations in *Rumex gmelini* seedlings after inoculated with *Fm* for 40, 60, and 80 days. This indicated the great potential value of *Fm* and *Pi* in promoting the resveratrol content of *P*. *cuspidatum*. In fact, in resveratrol-producing plants, the resveratrol biosynthesis is mainly derived from the phenylpropanoid pathway, involving phenylalanine ammonia lyase (PAL), cinnamic acid 4-hydroxylase, 4-coumaric acid: coenzyme A ligase, and stilbene synthase (Zhang et al., 2020). AM fungi such as *Fm* and *Paraglomus occultum* have been shown to up-regulate the expression of *PtPAL1* in *Poncirus trifoliata* (Zhang Y. C. et al., 2019; Tian et al., 2021). This suggests that symbiotic fungi can promote resveratrol production by up-regulating the expression of genes involved in resveratrol biosynthesis, but the exact mechanism has yet to be investigated.

In addition to resveratrol, endophytic fungal treatments also significantly increased chrysophanol content, with *Fm* + *Pi* presenting the best benefit. A study by Wang S. (2008) on *Ophiopogon japonicus* also showed that *Fm* significantly increased chrysophanol content by 238% over the non-inoculated control. Significantly elevated chrysophanol content was shown at 80 and 100 days after *Fm* inoculation of *Rumex gmelini* seedlings (Wang D., 2008). This suggested that plant-associated endophytic fungi have a strong stimulating effect on the chrysophanol production of *P*. *cuspidatum*.

Another anthraquinone component, emodin, was also affected by endophytic fungi: decrease by *Fm* and increase by *Pi* and *Fm* + *Pi*. Similarly, polydatin and physcion were also diversely affected by endophytic fungal inoculation: the decrease by *Pi* but the increase by both *Fm* and *Fm* + *Pi*. These results indicate that the induction of certain secondary metabolite depended on the species of endophytic fungi, but dual inoculation with *Fm* and *Pi* still presented a positive effect among the three fungal inoculations, indicating the benefits of dual inoculation for the production of medicinal components. In this study, root aloe-emodin concentration was inhibited by *Fm* and *Pi* singly or in combination. However, Guo et al. (2014) detected the presence of aloe-emodin in five endophytic fungal strains of *Rumex gmelini* Turcz. The exact mechanism regarding the inhibition of aloe-emodin in inoculated plants remains unclear and may be associated with the expression patterns of key genes in the metabolic pathway of anthraquinones and stilbene regulated by endophytic fungi, which requires further experimental verification.

## Conclusion

In short, *Fm* and *Pi* individually or in combination were able to colonize the roots of *P*. *cuspidatum*. The fungal colonization improved plant growth performance and root morphology, in which *Pi* presented relatively better role in plant growth response. On the other hand, these endophytic fungi also significantly increased most disaccharide contents, but decreased or did not alter most monosaccharide contents. *Fm* and *Pi* single or in combination increased the contents of most medicinal constituents, especially resveratrol and chrysophanol, where *Fm* + *Pi* presented a relatively higher profile. Further studies will analyze how endophytic fungi increase levels of resveratrol in *P*. *cuspidatum*, and how *Fm* and *Pi* synergically promote resveratrol production. Therefore, root-associated endophytic fungi can be introduced in the cultivation of *P*. *cuspidatum* to promote the production of some medicinal ingredients. In addition, further identification and screening of endophytic fungi in the field *P*. *cuspidatum* are needed.

## Data Availability Statement

The original contributions presented in the study are included in the article/supplementary material, further inquiries can be directed to the corresponding author.

## Author Contributions

R-TS and Q-SW designed the experiment. Z-ZZ and H-DF prepared the materials for the experiment. R-TS, X-CF, NZ, Y-ML, and Q-SW analyzed the data. R-TS wrote the manuscript. AH, A-BA-A, EA, and Q-SW revised the manuscript. All authors have read and agreed to the published version of the manuscript.

## Conflict of Interest

The authors declare that the research was conducted in the absence of any commercial or financial relationships that could be construed as a potential conflict of interest.

## Publisher’s Note

All claims expressed in this article are solely those of the authors and do not necessarily represent those of their affiliated organizations, or those of the publisher, the editors and the reviewers. Any product that may be evaluated in this article, or claim that may be made by its manufacturer, is not guaranteed or endorsed by the publisher.
